# Formation of fan psychological ownership and Its influence on fan enthusiasm: a hybrid approach using PLS-SEM and fsQCA

**DOI:** 10.3389/fpsyg.2025.1721382

**Published:** 2025-12-10

**Authors:** Qiuhao Huang, Yanxue Zou

**Affiliations:** School of Media and Communication, Shenzhen University, Shenzhen, China

**Keywords:** psychological ownership, fans, idols, fan enthusiasm, fandom economy

## Abstract

Driven by digital platforms and algorithmic ecosystems, contemporary fan culture has evolved into an unprecedented form of intensity and density. Fans willingly devote vast amounts of data labor, daily engagement in comment management, and financial voting to support their idols. Although prior research has primarily explained fan behavior through parasocial relationships or identity-based mechanisms, these approaches fall short of accounting for fans’ sovereignty-like sense of responsibility and sustained commitment. To address this gap, the present study introduces psychological ownership as a core explanatory lens and proposes a dual-pathway model consisting of idol–fan psychological ownership and fans’ collective psychological ownership to uncover the psychological drivers of fan enthusiasm in the digital era. Using a mixed-method design integrating Partial Least Squares Structural Equation Modeling (PLS-SEM) and fuzzy-set Qualitative Comparative Analysis (fsQCA), we analyzed survey data from 402 Chinese fans of fostered idols (Teens in Times and TFBOYS). PLS-SEM results indicate that self-investment, intimate interaction, and cultural symbol value positively contribute to the formation of both idol-fan psychological ownership and fans’ collective psychological ownership. In contrast, perceived control and fan emotionality predict only idol–fan psychological ownership, exerting no significant effect on collective ownership. Both forms of psychological ownership significantly enhance fan enthusiasm. Complementing these findings, fsQCA identified three distinct configurations—interaction-oriented, control-oriented, and emotion-driven patterns—that lead to high fan enthusiasm, revealing the causal complexity underlying fan behavior. This study extends psychological ownership theory into the domain of digital fan culture and enriches theoretical understandings at the intersection of fan studies and consumer behavior. The findings also offer important practical insights for idol-industry managers, brand strategists, and platform governance practitioners.

## 1 Introduction

The rise of consumer society has fostered a new emotional network centered around idols, transforming fans from passive consumers into active companions in the developmental journey of their idols (Zhao and Wu, 2021). The popular Chinese song “Chasing the Light” vividly captures this dynamic: “If you are the firework on the sea, I am the bubble of the waves; at that moment, your light illuminates me… I can follow behind you, like a shadow chasing the light in a dream.” In this metaphor, the “light” symbolizes the admired figure and the virtues embodied by that figure, whereas the “chaser of light” represents those who support, accompany, and protect the idol quietly yet steadfastly. The song offers a delicate portrayal of the follower’s inner world, illustrating a person who stays close to the idol like a shadow and quietly accompanies them. This imagery provides a vivid representation of the emotional connection that often defines contemporary idol-fan relationships.

In the context of participatory culture, the personal growth of idols is shaped, curated, and marketed to fans by management companies. As fans devote themselves to following and supporting their idols, they experience strong positive emotions, and their enthusiasm generates substantial commercial value (Xie and Lou, 2024). The popular expression “labor of love” reflects how fans willingly invest significant amounts of time, energy, and money to sustain their idols’ success, often with a sense of wholehearted commitment (Zhao and Wu, 2021). The intensity of this devotion brings forward an essential question: What motivates fans to make such profound emotional and financial investments in their idols?

In the study of digital fan culture, existing research on fan identity and parasocial relationships has illuminated the emotional bonds that develop between fans and idols (Liebers and Schramm, 2019; Forner et al., 2025). However, these perspectives remain insufficient for explaining the possessiveness, agency, and sense of responsibility that fans often display. This limitation becomes especially apparent in digital media environments, where emerging fan practices—such as affective labor (Wu et al., 2025) and data-based ranking support activities (He and Li, 2023; Wu et al., 2025)—show that fans are not only emotionally attached to their idols but also enact symbolic forms of ownership and responsibility through their actions and cognitive engagement (Dan et al., 2023; Xiao et al., 2025).

Research has increasingly highlighted the darker side of fan culture as well. Intense idol fixation and identity fusion may trigger problematic behaviors such as online aggression (Forner et al., 2025), emotional dysregulation (Leite et al., 2020), and toxic interactions within fandom communities (Proctor and Kies, 2018). These complex psychological mechanisms extend beyond traditional theories of attachment or identification and call for a more robust framework to explain why fans exhibit quasi-religious fervor and deep emotional investment in their idols.

To address this gap, the present study adopts psychological ownership theory as a central explanatory framework. According to this theory, individuals develop feelings of affection, protection, and possessiveness toward targets they perceive as “theirs” (Pierce et al., 2003). This lens provides a compelling basis for examining the formation of fan enthusiasm in contemporary digital fandom.

Furthermore, there is a clear gap between fan studies and research on psychological ownership. Existing work on psychological ownership has focused mainly on contexts such as social media (Kwon, 2020), brands and products (Kumar, 2019), and tourism (Qu et al., 2021), and has not yet been extended to the idol–fan domain. This gap becomes especially salient in the context of fostered idols. In such settings, fans are not only observers or consumers. They also act as co-creators in the idol’s development. Through financial contributions and emotional labor, fans participate in the entire process of an idol’s growth, from early obscurity to public recognition, and this participation provides an experience similar to co-producing a customized product. The sense of being an “owner” can activate feelings of responsibility, possessiveness, and protectiveness. These feelings help explain why fans are willing to devote significant time, money, and emotional energy to support their idols, and why they may experience strong negative emotions when this perceived ownership feels threatened.

At the same time, current research on psychological ownership shows limitations in three important respects. First, many studies overlook the role of target attributes (Krauss and Wienrich, 2025). Second, research has not sufficiently addressed individual differences, which leaves the heterogeneity among users unexplained (Peck and Luangrath, 2023). Third, most studies focus on ownership as a personal feeling that “this belongs to me” (Verkuyten, 2025), and pay much less attention to how individual and collective forms of psychological ownership may operate together. Recognizing these gaps helps clarify how psychological ownership contributes to fan identity formation and meaning-making.

In addition, fan behavior is shaped by multiple conditions, including emotional investment, group norms, symbolic identification, and algorithmic environments (Xiao et al., 2025). Many previous studies have relied on single-variable analytical approaches, which are insufficient for capturing the complex psychological and social mechanisms that jointly produce fan enthusiasm. For this reason, the present study combines Partial Least Squares Structural Equation Modeling (PLS-SEM) with fuzzy-set Qualitative Comparative Analysis (fsQCA). This mixed approach allows us to examine psychological ownership and fan enthusiasm among Chinese fans of fostered idols from both variable-centered and configuration-based perspectives.

Guided by the classic antecedents of psychological ownership, which include control, investment, and intimate knowing (Pierce et al., 2003), and informed by idol attributes and fan characteristics, this study focuses on five antecedent variables: perceived control, self-investment, intimate interactions, cultural symbol value, and fan emotionality. These variables are used to examine how both idol-fan psychological ownership and fans’ collective psychological ownership are formed. The study also investigates how these two forms of psychological ownership contribute to Fan enthusiasm. In this context, fan participation involves not only emotional engagement but also various forms of affective labor, which further highlights the relevance of psychological ownership in understanding fan behavior.

## 2 Theoretical framework

### 2.1 Psychological ownership

Pierce et al. (2003) first introduced the concept of psychological ownership in the field of organizational behavior, defining it as a psychological state in which individuals perceive a target object (whether material or immaterial) or part of it as “theirs,” fostering feelings of attachment and protection toward that object. Psychological ownership is characterized by three aspects: first, a sense of possession over a specific object; second, the relationship between the individual and the object, which is closely linked to the individual’s self-concept; and third, a complex psychological state that encompasses both cognitive and emotional components (Pierce et al., 2003; Van Dyne and Pierce, 2004). In recent years, this concept has expanded beyond organizational behavior and has been applied to consumer behavior, brand relationships, and digital media contexts, where it has become an important psychological mechanism for explaining loyalty, engagement, and protective behaviors (Reppmann et al., 2025).

Psychological ownership theory provides a framework with both cognitive and emotional explanatory power for understanding fan enthusiasm. In contrast to belongingness, which emphasizes relational connectedness, and identification, which highlights role-based affiliation, psychological ownership is distinctive because it elicits feelings of possessiveness and responsibility toward a target. These feelings motivate individuals to protect, invest in, and even defend the target. This mechanism means that individuals do not merely identify with the object; they also experience a sense of possessing it, which creates a deeper emotional and motivational bond that extends beyond formal or institutional affiliation (Avey et al., 2009; Brown et al., 2014). This perspective helps explain why fans often display intense passion and proactive behaviors. As consumers, fans function as “active audiences” and simultaneously resemble the “ownership customers” described in marketing research, whose strong sense of psychological ownership translates into substantial commercial value (Xie and Lou, 2024).

Building on this theoretical perspective, the present study introduces Kumar’s (2019) dual-dimension framework of individual and collective psychological ownership into the context of fan culture. This framework helps explain how fans develop possessive feelings toward idols as well as a shared sense of group ownership. Specifically, Idol-fan psychological ownership refers to a psychological state in which fans perceive their idol as an extension of the self, characterized by feelings of possessiveness, perceived influence, and emotional attachment. This sense of ownership often emerges from the fulfillment of personal motives, such as striving for an ideal self, enhancing positive self-esteem, or maintaining self-consistency (Lee and Suh, 2015; Kwon, 2020). Fans’ collective psychological ownership reflects the shared belief among fans that the ownership target belongs to the group, and that the idol is a collective object of care and responsibility. Together, these two dimensions form the structure of psychological ownership within the idol-fan domain. They explain symbolic possession and identification at the individual level, while also revealing collaboration and cohesion at the group level.

### 2.2 Motivation for psychological ownership

Pierce et al. (2001) argued that psychological ownership arises from three fundamental motives: efficacy, self-identity, and belongingness. They proposed that these motives can be fulfilled through three pathways: investing the self into the target, having control over the target, and intimately knowing the target. These pathways have been widely supported in various contexts such as virtual brand communities (Lee and Suh, 2015; Kumar, 2019), social media environments (Kwon, 2020), online games and virtual goods (Krauss and Wienrich, 2025).

Despite these advances, current psychological ownership research has tended to overlook the role of target attributes (Krauss and Wienrich, 2025). Pierce et al. (2003) noted that feelings of ownership can be shaped by an object’s appeal, accessibility, and manipulability. However, these object-related factors were not integrated into their core model of psychological ownership. In fan culture, idols carry highly symbolic and socially meaningful attributes. Their cultural symbolism not only strengthens identity-related motives but may also play a central role in triggering psychological ownership. For this reason, the present study introduces cultural symbol value as an antecedent variable to examine how the attributes of the ownership target can stimulate the emergence of ownership feelings in fan contexts.

In addition, recent studies have highlighted the importance of identity signaling and individual differences in the development of psychological ownership (Peck and Luangrath, 2023). For instance, research by Kirk et al. (2018) shows that individuals with narcissistic tendencies view ownership as a way to signal a superior self to others. They also display strong territorial behaviors in order to psychologically protect their identity. Fans often exhibit heightened emotional intensity and deep affective involvement, both of which can lead to territoriality and other irrational behaviors such as toxic interactions. However, existing research has not yet examined how fan characteristics influence psychological ownership.

Fan culture is shaped by both symbolic social meanings and strong emotional and interactive dynamics at the individual level. Building on this dual nature, the present study integrates the classic motivational pathways of psychological ownership (control, intimate knowing, and self-investment), the attribute dimension of the ownership target (cultural symbol value), and the individual characteristic dimension (fan emotionality). Based on these elements, we develop a psychological ownership formation framework suited to fan culture. This framework serves to reveal the mechanisms underlying idol-fan psychological ownership and fans’ collective psychological ownership, and it provides theoretical support for understanding fan enthusiasm, group collaboration, and sustained participation in the idol economy.

Perceived control is an important pathway in the formation of psychological ownership. When individuals feel that they can control a target, they are more likely to experience a sense of ownership toward it (Pierce et al., 2003; Peck and Luangrath, 2023). Research on psychological ownership in relation to dematerialized targets, such as social media platforms, virtual objects, and online games, shows that physical control is not necessary. A mere perception of control is sufficient to elicit feelings of ownership (Peck and Luangrath, 2023; Kwon, 2020; Krauss and Wienrich, 2025). In this study, perceived control refers to fans’ imagined ability to control their idols or symbols that represent their idols, such as behaviors, images, or public statements. Through this imagined control, fans feel that they can subjectively reduce the distance between themselves and the idol.

Psychological ownership is different from legal ownership. Individuals may experience a sense of ownership toward targets that they do not legally possess (Pierce et al., 2003). Although fans cannot legally own idols, nor can they fully control or directly interact with them in reality, they can abstract idols into symbolic forms carrying different meanings. Through media stimuli and participatory activities, fans perceive that they can influence these symbolic targets, for example through secondary creations and fan-made content (Tian and Adorjan, 2016). Kwon’s (2020) research on social media users further shows that perceived control has a positive effect on psychological ownership. Therefore, we propose the following hypothesis:

*H1: Perceived control has a positive effect on idol-fan psychological ownership.*

Digital platforms provide users with unprecedented space for symbolic manipulation. For example, in 2018, activists coordinated efforts to influence Google’s search ranking algorithm so that the term “idiot” became strongly associated with images of Donald Trump (Haynes, 2018). This kind of collective manipulation of public representations strengthens users’ perceived control over the target.

Kumar (2019) also pointed out that when individuals in a community experience a shared sense of control, joint investment, and shared autonomy, psychological ownership can extend from the individual level to the group level, resulting in stable collective psychological ownership. Similar to brand communities, digital fan communities are organized around a strong sense of “we,” shared narratives, and collective actions. Through activities such as collective voting, coordinated comment management, defending idols, and unified support practices, fans experience a shared sense of control over the idol’s development, especially within the visualized feedback mechanisms created by platform algorithms (Pierce et al., 2018; Xiao et al., 2025). Therefore, we propose the following hypotheses:

*H2: Perceived control has a positive effect on fans’ collective psychological ownership.*

Self-investment refers to the extent to which individuals devote their time, thoughts, skills, physical effort, psychological energy, and emotional resources to a target in order to develop a sense of belonging (Pierce et al., 2003). For fans, self-investment involves the merging of the self with the idol and the development of a sense of belonging that ranges from surface-level attraction to deep emotional involvement. Marketing research shows that self-investment is closely related to identity-based motives that promote psychological ownership (Pierce et al., 2003). Characteristics associated with the self, along with positive self-associations, are transferred to the target, which increases the perceived value of that target (Weiss and Johar, 2016). Idols provide fans with a channel to project their ideal self (idealized idol traits) onto their actual self (perceived similarity between the fan and the idol). This process narrows the gap between the ideal and actual self, reduces perceived self-discrepancy, strengthens identification, and facilitates the development of psychological ownership (Lee and Suh, 2015).

Furthermore, one of the most evident and influential forms of personal investment in a target is creation (Rudmin and Berry, 1987). Creation requires individuals to invest time, effort, and even personal values and identity. Fans engage in activities such as producing fan-made content, designing support slogans, and creating memes or other narrative elements that circulate in the public sphere. Through these practices, fans embed their own time, skills, aesthetics, and emotions into content related to the idol, leaving traces of self-extension within the idol’s public image (Zheng, 2023). In this way, fans use the ownership target to define their actual or desired self and to maintain self-consistency. Therefore, we propose the following hypotheses:

*H3: Self-investment has a positive effect on idol–fan psychological ownership.*

Self-investment not only shapes the dyadic relationship between the fan and the idol but also produces an accumulative effect at the group level. When fans create content, participate in support campaigns, or collaborate in data labor, they develop shared experiences, emotional resonance, and similar levels of self-discrepancy. These shared conditions foster mutual recognition within the fan community (Kumar, 2019; Zhang et al., 2023; Xiao et al., 2025). Therefore, we propose the following hypotheses:

*H4: Self-investment has a positive effect on fans’ collective psychological ownership.*

Intimate interactions refer to the psychological connections formed through exchanges between fans and idols, as well as among fans themselves. These interactions reduce perceived distance, create emotional resonance, and satisfy identification motives (Peck and Luangrath, 2023). Research on parasocial interaction shows that virtual interactions between fans and celebrities on social media influence fans’ quality of life and wellbeing (Kim and Kim, 2020). Other studies adopting a fan-celebrity relationship perspective also demonstrate that relationship involvement shapes how fans perceive their relationship with celebrities (Maheeya and Head, 2025). Therefore, idols’ social media activities, fan purchases of related merchandise, and participation in offline support events together constitute the foundation of intimate interactions between fans and idols. Through these experiences, fans deepen their emotional attachment, enhance identification, and develop a psychological sense of ownership. Therefore, we propose the following hypotheses:

*H5: Intimate interactions have a positive effect on idol-fan psychological ownership.*

At the group level, interaction is rooted in shared experiences and group-based identification. Through collective interaction, the ownership target becomes part of the group’s continued existence (Kišjuhas, 2024; Verkuyten, 2025). Zhu et al. (2013) also found that relational foundations and perceived closeness developed through social exchanges promote the formation of psychological ownership. Therefore, fans may not only experience intimacy with idols but also develop a sense of collective intimacy and shared ownership within the fan community. Therefore, we propose the following hypotheses:

*H6: Intimate interactions have a positive effect on fans’ collective psychological ownership.*

Cultural symbol value reflects the extent to which fans cognitively and affectively integrate idols into their everyday lives. Idols are not only consumed as media products but are also internalized as symbolic markers of identity. This symbolic mechanism shapes how fans construct meaning around idols and how they develop emotional identification with them (Pimentel and Reynolds, 2004).

Previous research has similarly emphasized the importance of symbolic factors in the formation of psychological ownership. Kumar and Kaushal (2021) noted that when a target carries symbolic identity functions, it is more likely to be incorporated into an individual’s self-concept, thereby evoking a sense of ownership. Symbolic objects can also serve compensatory functions when feelings of efficacy or belongingness are lacking. For example, consumers who experience social exclusion are more inclined to purchase products that symbolize group identity (DeWall and Richman, 2011). From the perspective of target attributes, Pick (2021) showed that symbolic cues associated with social media influencers—such as attractiveness, expertise, and credibility—strengthen consumers’ psychological ownership, brand attitudes, and purchase intentions. Similarly, Qu et al. (2021) found in their research on China’s national image and national identity that symbolic entities can elicit psychological ownership toward abstract targets. Therefore, individual fans interpret idols’ images, behaviors, and public narratives as part of their own identity system. Through this process, they develop cognitive and emotional bonds that reflect a sense of “this is my idol.” Therefore, we propose the following hypotheses:

*H7: Cultural symbol value has a positive effect on idol–fan psychological ownership.*

In digital fan culture, symbolic value is continuously reproduced through group symbols such as fan names, support colors, and internal discursive practices. For instance, the “four-leaf clover,” which symbolizes fans of TFBOYS, not only conveys emotional belonging but also demarcates the boundaries of the in-group. Fan names, support colors, and ritualized practices further contribute to a highly organized fandom structure that reinforces identity identification with both the idol and the fan community (Dan et al., 2023). Peck and Luangrath (2018) highlighted the positive halo effect of psychological ownership, suggesting that symbolic carriers such as idol merchandise or shared fan labels can evoke collective feelings of ownership. Accordingly, cultural symbol value strengthens the shared meanings within the group. Through ritualized interactions, support norms, and community-specific language, fans experience a sense of “we collectively own the idol.” Therefore, we propose the following hypotheses:

*H8: Cultural symbol value has a positive effect on fans’ collective psychological ownership.*

Fan emotionality refers to the distinctive emotional characteristics that fans display. In this study, fan emotionality is defined as the sensitivity, depth, and intensity of fans’ emotional responses toward idols. It involves both individual emotional traits and shared affective tendencies within the fan community, such as extroversion, enthusiasm, and energetic passion. Prior research has found that individuals with stronger emotionality and extroverted tendencies are more likely to develop intense celebrity worship and emotional attachment (McCarley and Escoto, 2003). Research on psychological ownership has also emphasized that ownership experiences are significantly shaped by emotional traits and personality variables (Peck and Luangrath, 2018). Therefore, we propose the following hypotheses:

*H9: Fan emotionality has a positive effect on idol-fan psychological ownership.*

Within fan culture, emotional sensitivity is not only an individual difference but also a mobilizable collective emotional resource. Grossberg (1992) argued that fans’ emotional sensitivity enables them to derive emotional energy and meaning from cultural texts and practices. Through cycles of investment and feedback, fans construct emotional structures that generate a sense of empowerment and identity. Reysen and Branscombe (2010) noted that even in the absence of formal organizational membership, fans tend to view themselves as part of an “imagined fan community,” forming group boundaries, in-group and out-group distinctions, and shared emotional reactions.

Furthermore, recent research on algorithmic emotionality suggests that fan mobilization is often driven not by rational benefits but by emotionally charged rhetoric, guilt-based narratives, and responsibility-oriented frames. Examples include appeals such as “only we have the idol” or “not contributing data is betrayal.” Fans with higher emotional sensitivity are more likely to be activated by these narratives. They experience stronger feelings of responsibility, urgency, and identity-based obligation, which deepen their emotional investment in the idol. This investment is then internalized as a symbolic sense of ownership toward the idol (Zhang et al., 2023; Xiao et al., 2025). Therefore, we propose the following hypotheses:

*H10: Fan emotionality has a positive effect on fans’ collective psychological ownership.*

### 2.3 Psychological ownership and fan enthusiasm

Enthusiasm is a psychological construct that reflects individuals’ passionate and regular engagement in activities. It serves as a foundation for fulfilling basic needs and creating a sense of pleasure, and it significantly influences psychological functioning (Baradar et al., 2023). Research on Fan enthusiasm originated in the field of sports. Drawing on sports fandom, Pimentel and Reynolds (2004) proposed that fans often develop a religious-like fervor and infatuation toward the celebrities they admire. This fervor is expressed through behaviors such as purchasing and collecting merchandise, sharing opinions, sacrificing personal time, and encouraging others to join the fan community. Pichler and Hemetsberger (2008) further described fan enthusiasm as characterized by devotion, intimacy, and passion. At this early stage, fan enthusiasm primarily centered on individual identification with and support for tangible objects such as teams or athletes. Its expression was largely constrained by the physical experience of being present in specific spaces, such as attending games or purchasing physical products.

With the rise of mobile internet and social media, fans can now participate in real-time narrative production and public opinion management surrounding their idols. Fan enthusiasm manifests not only as affection and consumption at the individual level but also as highly organized collective action. Examples include data-based ranking activities (He and Li, 2023; Wu et al., 2025; Zhang et al., 2023), comment management (Wu et al., 2025), fundraising for support events, and symbolic labor (Zheng, 2023). Through platform algorithms, fans actively shape the idol’s public image and social value, transforming emotional investment into quantifiable metrics such as super-topic levels, repost and comment counts, and ranking positions. These quantifiable outcomes, in turn, generate recognition and a sense of achievement (Zhang et al., 2023; Xiao et al., 2025).

Therefore, fan enthusiasm enhances individual consumption willingness and constructs a more stable consumption motivation mechanism through collective identity formation. This shift indicates that fan enthusiasm has evolved from traditional individualized emotional identification to platform-driven collective participation within a broader emotional economy. Unlike conventional conceptualizations of fan loyalty, which emphasize repeated purchasing, fan enthusiasm highlights emotional depth, group identity, and sustained self-investment. It represents a long-term state characterized by both emotional intensity and behavioral engagement (Pimentel and Reynolds, 2004; Baradar et al., 2023).

Research on possessiveness shows that ownership objects and ownership perceptions activate self-target connections, a sense of responsibility, and positive attitudes, which motivate individuals to protect the ownership target, evaluate it favorably, and even make sacrifices on its behalf (Pierce et al., 2001; Peck and Luangrath, 2023). Psychological ownership also generates a series of positive effects, including enhanced well-being, self-esteem, and identity, which extend to other objects associated with the ownership target. For example, stronger psychological ownership toward an idol can translate into behaviors such as purchasing related merchandise, participating in collective activities, and disseminating idol-related content. Therefore, we propose the following hypotheses:

*H11: Idol-fan psychological ownership has a positive effect on fan enthusiasm.*

Research in organizational behavior has shown that employees who experience psychological ownership toward their organizations tend to display positive attitudes, a strengthened self-concept, and a heightened sense of responsibility, all of which promote organizational commitment (Van Dyne and Pierce, 2004). Other studies have found that members who feel psychological ownership toward a community develop broad motivational tendencies and are likely to exhibit behaviors characterized by dedication, responsibility, and enthusiasm (Hou and Zhang, 2021). As specialized consumers, fans may also be influenced by such positive collective cognitions and emotions.

Recent research on fan behavior indicates that under the influence of algorithmic recommendation and data visualization, fan enthusiasm has become highly visible, responsive to immediate feedback, and shaped by group negotiation. Fans strategically participate in platform algorithms and engage in collective algorithmic actions to strengthen their influence and enhance the visibility of their idols (Xiao et al., 2025). Therefore, we propose the following hypotheses:

*H12: Fans’ collective psychological ownership has a positive effect on fan enthusiasm.*

### 2.4 The mediating role of psychological ownership

In the context of digital fan culture, fans’ identification with idols, their sense of participation, and their emotional bonds can also generate favorable cognitions and emotions, which enhance support behaviors, data-related labor, and sustained participation (Zhang et al., 2023). These observations suggest that psychological ownership motives play a positive role in shaping idol–fan relationships, influencing fans’ psychological perceptions of both idols and the fan community, and further driving enthusiastic behaviors. Therefore, we propose the following hypotheses:

*H13: Idol–fan psychological ownership mediates the effects of perceived control (a), self-investment (b), and intimate interactions (c) on fan enthusiasm.*

*H14: Fans’ collective psychological ownership mediates the effects of perceived control (a), self-investment (b), and intimate interactions (c) on fan enthusiasm.*

*H15: Idol–fan psychological ownership mediates the effects of cultural symbol value (a) and fan emotionality (b) on fan enthusiasm.*

*H16: Fans’ collective psychological ownership mediates the effects of cultural symbol value (a) and fan emotionality (b) on fan enthusiasm.*

### 2.5 Research propositions for fsQCA

Building on the multiple motives proposed in psychological ownership theory—efficacy, self-identity, and belonging (Pierce et al., 2003)—as well as the emotional intensity and diverse drivers that characterize fan behavior (Pimentel and Reynolds, 2004; Arvidsson and Caliandro, 2016; Zhao and Wu, 2021), we assume that fan enthusiasm is unlikely to arise from a single factor. Instead, fans may display high enthusiasm through different combinations of conditions that lead to the same outcome. Thus, we expect the fsQCA to identify multiple configurations that can all produce high fan enthusiasm, rather than revealing any single necessary condition.

*P1: The formation of fan enthusiasm will exhibit equifinality, with multiple pathways leading to the same outcome.*

Moreover, research on parasocial interaction, social identity, and celebrity worship (Liebers and Schramm, 2019; Forner et al., 2025; Tajfel and Turner, 1979; Pimentel and Reynolds, 2004) indicates that fan behaviors are jointly shaped by interactional relationships, symbolic meanings, and individual emotionality. Some of these factors may serve as functional substitutes for one another. Accordingly, we anticipate that the fsQCA will show that certain antecedents, although non-significant in a single linear model, become influential when combined with other conditions. This reflects causal complexity, in which different configurations compensate for or complement one another in producing high fan enthusiasm. Based on this reasoning, we propose:

*P2: The antecedent variables will display configurational substitutability, with different factors acting as complements or substitutes across different pathways.*

Finally, the central role of psychological ownership in fan behavior suggests that it may appear repeatedly as a key condition across multiple configurations. Idol–fan psychological ownership and fans’ collective psychological ownership represent individual-level and group-level possessive engagement, respectively. Both have been shown to strengthen identification, responsibility, and sustained action tendencies (Pierce et al., 2018; Peck and Luangrath, 2023). Therefore, we propose the following integrative proposition linking the PLS-SEM and fsQCA results:

*P3: The fsQCA configurations leading to high fan enthusiasm will require the presence of the core mechanisms identified by the PLS-SEM model. Specifically, we expect idol–fan psychological ownership and fans’ collective psychological ownership—both central mediators in the PLS-SEM—to emerge as core conditions in the fsQCA configurations associated with high enthusiasm.*

**FIGURE 1:**
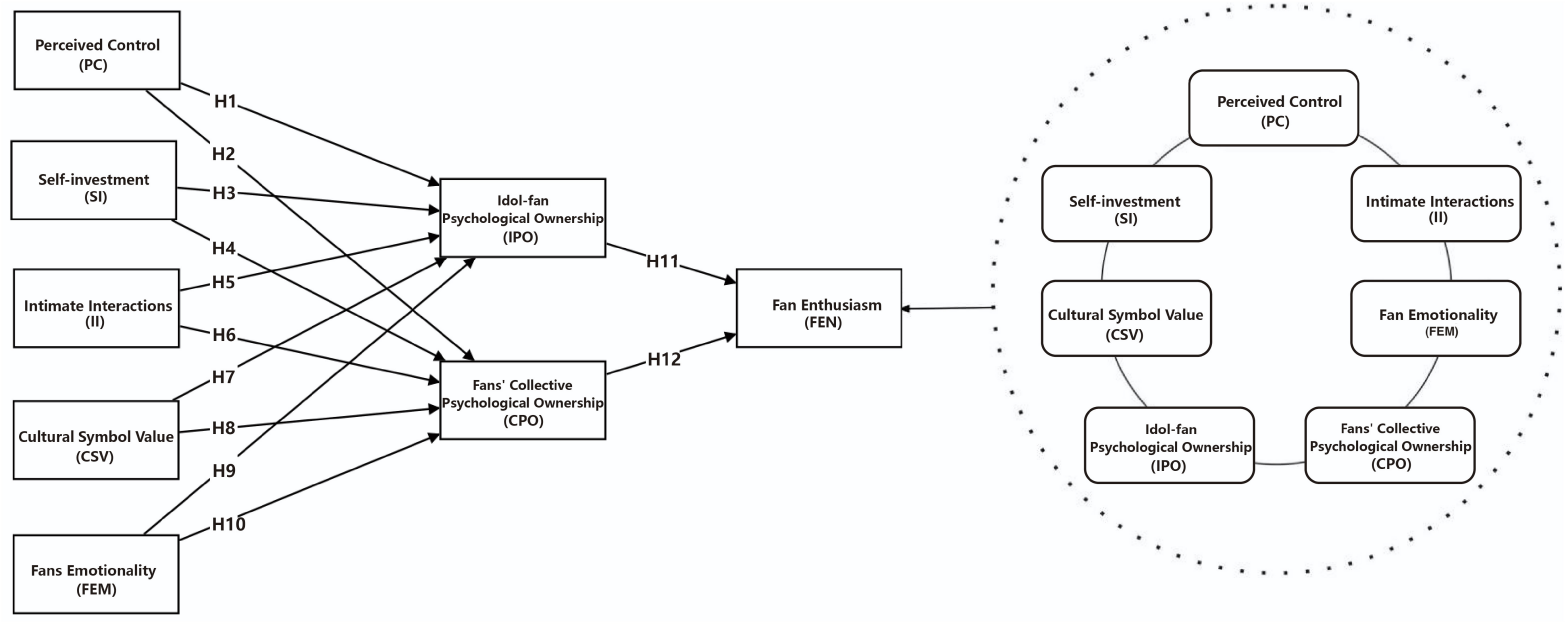
Research hypothesis and propositions model.

Our research model is presented in Figure 1. The left side illustrates the PLS-SEM model, and the right side depicts the fsQCA model.

## 3 Materials and methods

### 3.1 Questionnaire design

This study employed a questionnaire composed of two sections. The first section included questions designed to collect respondents’ demographic information. The second section consisted of 29 items used to measure the eight variables presented in Figure 1. All measurement scales have demonstrated strong reliability and validity in prior research and are therefore appropriate for the present study. These scales include: self-investment (Reysen and Branscombe, 2010), perceived control (Kwon, 2020), intimate interactions (Kwon, 2020), cultural symbol value (McCutcheon et al., 2002; Stokburger-Sauer et al., 2012), fan emotionality (Schramm and Hartmann, 2008), idol–fan psychological ownership and fans’ collective psychological ownership (Avey et al., 2009; Kumar, 2019), and fan enthusiasm (Pimentel and Reynolds, 2004; Baradar et al., 2023). All items were rated on a five-point Likert scale ranging from “1: strongly disagree” to “5: strongly agree.”

All scales used in this study were adapted from established English instruments. To ensure accurate comprehension among Chinese participants, all measurement items were translated into Chinese and adapted to the idol–fan context. We followed a translation–back-translation process, expert review procedures, and wording adjustments based on a pilot test to ensure cultural and contextual appropriateness for Chinese respondents.

According to the researchers’ pilot testing, participants required approximately 3–5 min to complete the questionnaire, and they were informed that the survey would take no longer than 5 min. The questionnaire also included a screening question to determine whether respondents identified as fans of Teens in Timesor TFBoys. Only those who confirmed fandom status were permitted to proceed. An instructional manipulation check (IMC) item was included as an attention check. The instruction stated: “To ensure you are answering carefully, please select “strongly disagree” for this item,” with response options: strongly agree / agree / neutral / disagree / strongly disagree.

To ensure data quality, non-response bias was assessed. The results indicated no significant differences in variable means between early and late respondents, suggesting that non-response bias was not a concern in this study (Armstrong and Overton, 1977).

### 3.2 Data collection

Data were collected using the online survey platform Wenjuanxing^1^. Fans of Teens in Times and TFBoys were randomly recruited as target respondents. The survey link was distributed primarily through Weibo and Tieba fan communities, as well as WeChat fan groups, where fans were invited to participate voluntarily.

The survey was administered anonymously beginning on 11 November 2024, and data collection was completed on 23 November 2024. After removing invalid responses—such as those with completion times under 1 min, highly uniform or patterned answering, or failure to pass the attention check—a total of 402 valid questionnaires were retained for analysis. All collected information was anonymized to prevent privacy risks, and participants were fully informed of these measures. The demographic details of the sample are presented in Table 1.

**TABLE 1:** Respondents’ demographic profiles (*N* = 402).

Item	Category	Frequency	Percentage
Gender	Male	175	43.5%
	Female	227	56.5%
Age	18 or younger	10	2.5%
	19–29	228	56.7%
	30–39	90	22.4%
	40–49	51	12.7%
	50 or older	23	5.7%
Education	High school education or less	32	8.0%
	Associate degree	47	11.7%
	Bachelor’s degree	271	67.4%
	Master’s degree or higher	52	12.9%

### 3.3 Data analysis

This study employed PLS-SEM to analyze the sample data and assess both the measurement and structural models (Hair et al., 2019). fsQCA was then used to identify combinations of conditions that lead to the outcome of interest (Ragin, 2008).

Compared with other SEM techniques, PLS-SEM is well suited for predicting complex models and can accommodate non-normal data distributions and relatively small samples (Hair et al., 2019). The decision to use PLS-SEM in this study was based on several considerations. First, the exploratory nature of the research aligns with the strengths of PLS-SEM, as the study seeks to identify the antecedents and outcome structures of psychological ownership (Hair et al., 2017). Second, the proposed model includes eight constructs and multiple indicators, and PLS-SEM is particularly effective for analyzing such complex model structures (Hair et al., 2012). Third, although the sample size in this study is modest, it satisfies the minimum requirements for PLS-SEM, which recommend a sample size at least 10 times the largest number of structural paths directed at any endogenous construct (Hair, 2014). Fourth, PLS-SEM provides symmetric net effect analysis, allowing the assessment of both direct and indirect effects of each predictor on the outcome variable (Hair et al., 2017). Based on these considerations and methodological recommendations, SmartPLS 4.0 was used to conduct the PLS-SEM analysis in this study.

Subsequently, fsQCA was conducted to provide an additional analytic perspective that complements the PLS-SEM results. Because PLS-SEM is based on regression analysis, it focuses on the net effects of variables and assumes causal symmetry, which limits its ability to capture the complex causal patterns that arise from interdependent antecedent conditions (Pappas and Woodside, 2021). In contrast, fsQCA is well-suited for examining how combinations of independent variables jointly lead to an outcome. It offers advantages in handling causal asymmetry and configurational or combined effects (Fiss, 2011; Ragin, 2008).

As a set-theoretic approach, fsQCA can uncover the complexity of causal linkages among variables (Rihoux, 2006). The method has frequently been used to supplement findings from research models initially tested with SEM. For this study, fuzzy-set qualitative comparative analysis was employed to generate additional insights that complement the PLS-SEM findings and to verify the three research propositions proposed earlier. These analyses help provide a more comprehensive understanding of how psychological ownership shapes fan enthusiasm.

### 3.4 Common method bias

Because this study relied on self-reported data collected through a cross-sectional design and from a relatively single source, common method bias (CMB) may be a concern and could potentially threaten the reliability of the findings (Podsakoff et al., 2003). To mitigate this risk, two primary strategies were implemented: procedural design and statistical control.

On the procedural side, a pilot test was conducted and respondents were assured anonymity to reduce evaluation apprehension and response bias. On the statistical side, Harman’s one-factor test was performed. An unrotated factor analysis including all measurement items was conducted with the number of factors fixed to one. The single factor accounted for 42.757% of the total variance, below the commonly adopted 50% criterion (MacKenzie et al., 2005), suggesting that common method bias is not a serious concern in the present study.

## 4 Results

### 4.1 Measurement model

The PLS-SEM analysis followed the two-step procedure recommended by Hair et al. (2019), which involves evaluating the measurement model and then assessing the structural model. The measurement model was examined to determine the reliability and validity of the scales, and the structural model was evaluated to test the hypothesized relationships.

As shown in Table 2, both composite reliability and Cronbach’s α values for all constructs exceeded the recommended threshold of 0.70, indicating satisfactory reliability (Hair et al., 2019). The average variance extracted (AVE) values were all greater than 0.50, which suggests that each construct explains more than half of the variance of its indicators and therefore demonstrates adequate convergent validity (Hair et al., 2019; Fornell and Larcker, 1981).

**TABLE 2:** Reliability and validity of variables.

Variable	Items	Estimate	Cronbachs α	CR	AVE
Self-investment (SI)	SI1 I invest a great deal of time and emotional energy in my idol.	0.850	0.737	0.845	0.646
	SI2 I actively create or share content for my idol.	0.792			
	SI3 I am willing to sacrifice some of my personal time or money to support my idol.	0.766			
Perceived control (PC)	PC1I feel that I can influence my idol’s activities or career direction.	0.811	0.757	0.861	0.673
	PC2 When I participate in voting or support projects, I genuinely feel that my efforts make a difference.	0.824			
	PC3 Through interactions or comments, I feel that my idol can “hear my voice.”	0.826			
Intimate interactions (II)	II1 I frequently interact with my idol or the fan community through social media.	0.826	0.703	0.835	0.628
	II2 I feel that my idol is sincere when communicating with fans.	0.728			
	II3 I am willing to spend time interacting with my idol and related fan groups.	0.820			
Cultural symbol value (CSV)	CSV1 My idol represents a lifestyle or set of values that I identify with.	0.813	0.822	0.882	0.651
	CSV2 I like expressing my individuality by supporting my idol.	0.788			
	CSV3 My idol’s image reflects my ideal self or life aspirations.	0.801			
	CSV4 I feel a strong sense of identification with the idol I admire.	0.824			
Fan emotionality (FEM)	FEM1 I feel emotionally excited when my idol posts new updates.	0.794	0.815	0.878	0.644
	FEM2 I am easily influenced by my idol’s emotions or expressions.	0.823			
	FEM3 My idol’s success or failure strongly affects my mood.	0.814			
	FEM4 I would stand up for my idol without hesitation.	0.777			
Idol-fan psychological ownership (IPO)	IPO1 I feel as if the relationship between me and my idol is like that of family.	0.740	0.819	0.881	0.649
	IPO2 I feel a sense of responsibility for my idol’s actions.	0.856			
	IPO3 My idol’s success makes me feel proud, almost as if it were my own.	0.860			
	IPO4 In some sense, I feel that my idol belongs to me.	0.761			
Fans’ collective psychological ownership (CPO)	CPO1 I feel that I am part of the fan community, and together we protect our idol’s image.	0.737	0.811	0.876	0.639
	CPO2 I am willing to work hard for the honor of the fan group.	0.833			
	CPO3 I feel that our idol belongs to us as a fan community.	0.813			
	CPO4 When outsiders criticize my idol, I feel as if they are attacking “our” group.	0.811			
Fan enthusiasm (FEN)	FEN1 Supporting my idol fills me with passion and motivation.	0.861	0.871	0.912	0.721
	FEN2 My idol is one of the most important interests in my life.	0.828			
	FEN3 I encourage people around me to follow the idol I like.	0.861			
	FEN4 I plan to continue following and supporting my idol in the future, and my enthusiasm is unlikely to fade over time.	0.847			

CR, composite reliability; AVE, average variance extracted.

As presented in Table 3, the Fornell–Larcker criterion shows that the square root of each construct’s AVE (represented on the diagonal) is greater than its inter-construct correlations (represented off the diagonal), confirming discriminant validity (Fornell and Larcker, 1981). Additionally, Table 4 reports the heterotrait–monotrait ratio of correlations (HTMT), with all values below the 0.90 threshold, further supporting discriminant validity among the constructs (Hair et al., 2019). Taken together, all criteria indicate that discriminant validity was adequately established.

**TABLE 3:** Discriminant validity (Fornell-Larcker criterion).

Variable	II	IPO	PC	CSV	FEM	FEN	CPO	SI
II	**0.799**	–	–	–	–	–	–	–
IPO	0.718	**0.807**	–	–	–	–	–	–
PC	0.691	0.717	**0.849**	–	–	–	–	–
CSV	0.471	0.461	0.425	**0.802**	–	–	–	–
FEM	0.623	0.675	0.673	0.476	**0.793**	–	–	–
FEN	0.725	0.724	0.716	0.557	0.642	**0.806**	–	–
CPO	0.516	0.514	0.498	0.634	0.494	0.655	**0.820**	–
SI	0.644	0.652	0.678	0.363	0.542	0.641	0.445	**0.803**

The values on the diagonal are the square root of AVE. II, intimate interactions; IPO, idol-fan psychological ownership; PC, perceived control; CSV, Cultural symbol value; FEM, fan emotionality; FEN, fan enthusiasm; CPO, fans’ collective psychological ownership; SI, self-investment.

**TABLE 4:** Heterotrait-monotrait ratio (HTMT).

Variable	CPO	CSV	FEN	FEM	II	IPO	PC	SI
CPO	–	–	–	–	–	–	–	–
CSV	0.869	–	–	–	–	–	–	–
FEN	0.814	0.834	–	–	–	–	–	–
FEM	0.577	0.567	0.504	–	–	–	–	–
II	0.819	0.875	0.858	0.621	–	–	–	–
IPO	0.883	0.862	0.84	0.687	0.840	–	–	–
PC	0.659	0.651	0.615	0.805	0.676	0.835	–	–
SI	0.791	0.776	0.812	0.452	0.709	0.769	0.574	–

CPO, fans’ collective psychological ownership; CSV, cultural symbol value; FEN, fan enthusiasm; FEM, fan emotionality; II, intimate interactions; IPO, Idol-fan psychological ownership; PC, perceived control; SI, self-investment.

### 4.2 Structural model

Before analyzing the path coefficients, multicollinearity was assessed. PLS evaluates multicollinearity using the variance inflation factor (VIF), and values below 5 indicate no multicollinearity concerns (Kock, 2015). In this study, the highest VIF value was 2.444, suggesting that multicollinearity was not an issue. Model fit was assessed using the standardized root mean square residual (SRMR). An SRMR value of 0.08 or lower is considered acceptable (Henseler et al., 2015). The SRMR for the present model was 0.071, indicating adequate model fit.

**FIGURE 2:**
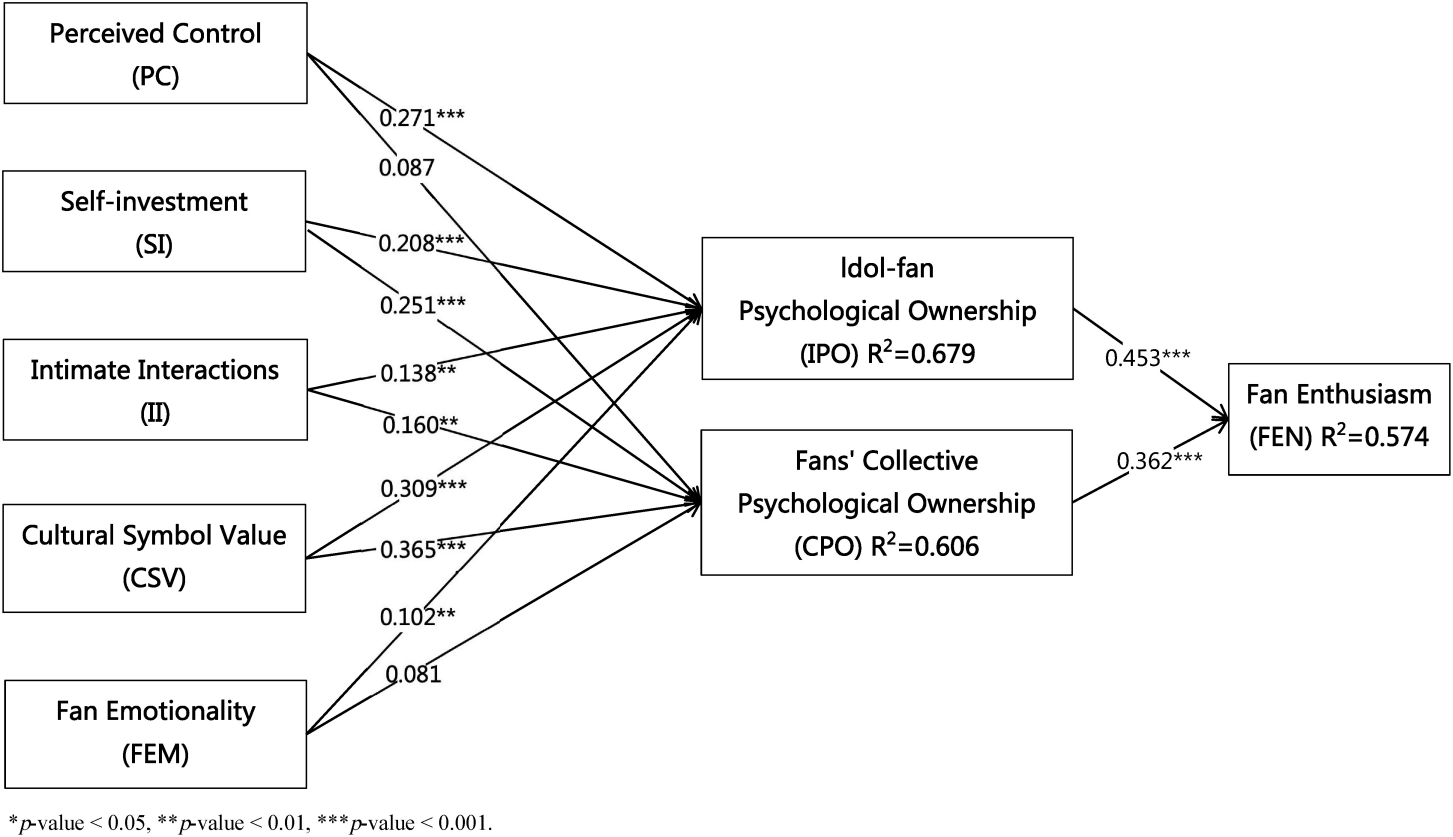
Results of the structural model.

Bootstrapping was performed with 5,000 subsamples. The standardized path coefficients and significance levels are presented in Figure 2 and Table 5. First, perceived control (β = 0.271, *p* < 0.001), self-investment (β = 0.208, p < 0.001), intimate interactions (β = 0.138, *p* < 0.01), cultural symbol value (β = 0.309, *p* < 0.001), and fan emotionality (β = 0.102, *p* < 0.01) all had positive effects on idol-fan psychological ownership. Thus, H1, H3, H5, H7, and H9 were supported.

**TABLE 5:** Results of the path analysis.

Hypothesis	Path	Path coefficients	STD	*P*	*T*	Support
H1	PC→IPO	0.271***	0.061	0.000	4.409	Yes
H2	PC→CPO	0.087	0.052	0.097	1.658	No
H3	SI→IPO	0.208***	0.039	0.000	5.304	Yes
H4	SI→CPO	0.251***	0.046	0.000	5.495	Yes
H5	II→IPO	0.138**	0.047	0.003	2.947	Yes
H6	II→CPO	0.160**	0.051	0.002	3.158	Yes
H7	CSV→IPO	0.309***	0.050	0.000	6.145	Yes
H8	CSV→CPO	0.365***	0.058	0.000	6.273	Yes
H9	FEM→IPO	0.102**	0.039	0.009	2.623	Yes
H10	FEM→CPO	0.081	0.043	0.063	1.860	No
H11	IPO→FEN	0.453***	0.069	0.000	6.534	Yes
H12	CPO→FEN	0.362***	0.069	0.000	5.207	Yes

**p-value < 0.01,

***p-value < 0.001. PC, perceived control; SI, self-investment; II, Intimate.

Second, self-investment (β = 0.251, *p* < 0.001), intimate interactions (β = 0.160, *p* < 0.01), and cultural symbol value (β = 0.365, *p* < 0.001) significantly influenced fans’ collective psychological ownership. Therefore, H4, H6, and H8 were supported. The effects of perceived control (β = 0.087, *p* > 0.05) and fan emotionality (β = 0.081, *p* > 0.05) on fans’ collective psychological ownership were not significant, so H2 and H10 were not supported.

Finally, idol-fan psychological ownership (β = 0.453, *p* < 0.001) and fans’ collective psychological ownership (β = 0.362, *p* < 0.001) positively predicted fan enthusiasm, providing support for H11 and H12.

In PLS-SEM, R^2^ indicates the amount of variance explained in each endogenous construct and serves as an important indicator of model explanatory power. As a general guideline, R^2^ values of 0.75, 0.50, and 0.25 can be considered substantial, moderate, and weak, respectively (Hair et al., 2019). As shown in Table 6, the R^2^ values for idol–fan psychological ownership (0.679), fans’ collective psychological ownership (0.606), and fan enthusiasm (0.574) indicate that the model demonstrates satisfactory explanatory power.

**TABLE 6:** Predictive relevance analysis.

Variable	R^2^	Q^2^
IPO	0.679	0.665
CPO	0.606	0.590
FEN	0.574	0.591

IPO, idol-fan psychological ownership; CPO, Fans’ collective psychological ownership; FEN, fan enthusiasm.

The blindfolding procedure was used to calculate Stone–Geisser’s Q^2^ value, which assesses the predictive relevance of a PLS path model. As shown in Table 6, the results show Q^2^ values of 0.665 for idol–fan psychological ownership, 0.590 for fans’ collective psychological ownership, and 0.591 for fan enthusiasm. All values exceed zero (Tenenhaus et al., 2005), indicating that the proposed model has strong predictive relevance for the reflective endogenous constructs.

In addition to examining the R^2^ values of all endogenous constructs, the model was also evaluated using effect size *f*^2^. The *f*^2^ analysis provides insight into the relative contribution of each exogenous construct to the model, complementing the overall significance of the relationships (Hair et al., 2019). Effect sizes can be interpreted as small (0.02 ≤ *f*^2^ < 0.15), medium (0.15 ≤ *f*^2^ < 0.35), and large (*f*^2^ ≥ 0.35) (Cohen, 2013).

For idol-fan psychological ownership, perceived control (*f*^2^ = 0.119), self-investment (*f*^2^ = 0.073), intimate interactions (*f*^2^ = 0.029), and cultural symbol value (*f*^2^ = 0.122) exhibited small effect sizes. For fans’ collective psychological ownership, self-investment (*f*^2^ = 0.087), intimate interactions (*f*^2^ = 0.032), and cultural symbol value (*f*^2^ = 0.138) also showed small effects. For fan enthusiasm, idol–fan psychological ownership (*f*^2^ = 0.228) and fans’ collective psychological ownership (*f*^2^ = 0.146) demonstrated medium effect sizes. All remaining paths showed negligible effects.

SmartPLS 4.0 allows direct testing of mediation effects using the bootstrapping procedure. In this study, mediation was assessed with 5,000 bootstrap samples. The results, presented in Table 7, show that all mediation effects were significant except for the indirect effects of perceived control and fan emotionality on fan enthusiasm through fans’ collective psychological ownership. Therefore, H13 and H15 were supported, whereas H14 and H16 received partial support.

**TABLE 7:** Mediating effects test results.

Path	Path coefficients	*P*	*T*	Support
PC→IPO→FEN	0.123***	0.000	5.197	Yes
PC→CPO→FEN	0.031	0.102	1.634	No
SI→IPO→FEN	0.094***	0.000	3.778	Yes
SI→CPO→FEN	0.091***	0.001	3.410	Yes
II→IPO→FEN	0.063*	0.018	2.377	Yes
II→CPO→FEN	0.058**	0.010	2.567	Yes
CSV→IPO→FEN	0.140***	0.000	4.086	Yes
CSV→CPO→FEN	0.132***	0.000	3.946	Yes
FEM→IPO→FEN	0.046*	0.020	2.330	Yes
FEM→CPO→FEN	0.029	0.086	1.715	No

*P-value < 0.05,

**p-value < 0.01,

***p-value < 0.001. PC, perceived control; SI, self-investment; II, intimate interactions; CSV, cultural symbol value; FEM, fan emotionality; FEN, fan enthusiasm; IPO, idol-fan psychological ownership; CPO, fans’ collective psychological ownership.

### 4.3 Fuzzy set qualitative comparative analysis (fsQCA)

First, seven variables were selected as antecedent conditions for the fsQCA: perceived control, self-investment, intimate interactions, cultural symbol value, fan emotionality, idol–fan psychological ownership, and fans’ collective psychological ownership. The sample size of this study (*N* = 402) meets the diversity requirements for medium-to-large sample fsQCA. Unlike traditional regression analysis, fsQCA does not rely on parameter estimation but instead focuses on case coverage and the explanatory power of condition configurations. With seven conditions, the sample demonstrates sufficient configurational diversity to identify robust solutions (Ragin, 2008; Greckhamer et al., 2018).

Before conducting the fsQCA, an essential step is data calibration. Calibration transforms raw data into fuzzy-set membership scores ranging from 0.0 to 1.0 (Ragin, 2008). Following Ragin’s (2008) direct calibration method, and based on the distribution of each variable, full membership was set at the 95th percentile, the crossover point at the 50th percentile, and full non-membership at the 5th percentile. The calibrated data are presented in Table 8.

**TABLE 8:** Data calibration.

Variable	Full non-membership (5%)	Crossover point (50%)	Full membership (95%)
SI	5.000	3.383	2.000
PC	5.000	4.000	2.666
II	5.000	4.000	2.666
CSV	5.000	4.250	2.250
FEM	5.000	4.250	2.500
IPO	5.000	4.000	2.750
CPO	5.000	4.000	2.500
FEN	5.000	4.000	2.250

SI, self-investment; PC, perceived control; II, intimate interactions; CSV, cultural symbol value; FEM, fan emotionality; IPO, idol-fan psychological ownership; CPO, fans’ collective psychological ownership; FEN, fan enthusiasm.

Second, a necessity analysis was conducted for each antecedent condition. The results showed that the consistency levels for all antecedent variables were below 0.90, indicating that no single condition was necessary for producing fan enthusiasm. This suggests that no individual antecedent alone can lead to the outcome, and that configurational analysis is required to identify the combinations of conditions that generate fan enthusiasm.

Before the sufficiency analysis, a truth table with 2K rows was constructed, where K represents the number of antecedent conditions. Each row represents a possible configuration of antecedents. Given that the sample size exceeds 150, the thresholds were set according to the recommendations of Fiss (2011): a frequency threshold of 3 cases, a consistency threshold of 0.90, and a PRI consistency threshold of 0.85. Based on these parameters, complex, parsimonious, and intermediate solutions were generated. Using the results from the intermediate and parsimonious solutions, configurations were identified and compared. The configurational results are presented in Table 8.

As shown in Table 9, the overall solution consistency is 0.948, which exceeds the established threshold. The overall coverage is 0.669, indicating that 66.9% of the cases are accounted for by the configurational solutions, suggesting strong explanatory power for the outcome. A comparative analysis across configurations reveals three distinct patterns that lead to high fan enthusiasm.

**TABLE 9:** Configurations for fan enthusiasm.

Antecedent factors	S1			S2	S3
	S1a	S1b	S1c		
SI	●	●	●	–	○
PC	○	○	○	●	○
II	●	●	●	●	–
CSV	–	⊗		○	○
FEM	⊗	–	○	–	●
IPO	○	○	–	●	●
CPO	–	–	○	●	●
Consistency	0.971	0.969	0.969	0.964	0.965
Coverage	0.317	0.316	0.476	0.580	0.499
Raw unique	0.002	0.015	0.012	0.080	0.034
Solution consistency	0.948				
Solution coverage	0.669				

●, core conditions; ○, peripheral conditions; ⊗, no path; SI, self-investment; PC, perceived control; II, intimate interactions; CSV, cultural symbol value; FEM, fan emotionality; IPO, idol-fan psychological ownership; CPO, fans’ collective psychological ownership.

Configuration S1: Interaction-oriented pattern. The core conditions of this configuration are high self-investment and high intimate interactions, with perceived control serving as a peripheral condition. This configuration includes three sub-configurations. Fans in this pattern develop psychological ownership by investing significant time and effort (self-investment) and by engaging in frequent interactions (intimate interactions), through which they accumulate deep, sustained knowledge about their idol.

Configuration S2: Control-oriented pattern. The core conditions include high perceived control, high intimate interactions, high idol–fan psychological ownership, and high fans’ collective psychological ownership. This configuration demonstrates high consistency (0.964) and accounts for the largest proportion of cases (coverage = 0.580). It commonly appears in highly organized and structured fan-support cultures. In this pattern, fan enthusiasm is driven primarily by the anticipated sense of influence over outcomes and the perceived effectiveness of collective action.

Configuration S3: Emotion-driven pattern. The core conditions are high fan emotionality, high idol–fan psychological ownership, and high fans’ collective psychological ownership. This configuration suggests that digital interactions foster new forms of intimacy. Fans with differing personalities and values may display fluctuating relational dynamics, and their enthusiasm is often shaped by strong emotional responsiveness and a tendency toward affect-driven or less rational reactions.

## 5 Conclusion and implications

### 5.1 Discussion of the results

With the continuous strengthening of platform algorithms, data visualization mechanisms, and collaborative fan norms, contemporary fan culture has developed into an intensely concentrated and highly organized landscape of enthusiasm. Fans participate in idol-related competition not only by generating data, managing comment sections, and engaging in paid voting, but also by continually investing emotional, temporal, and financial resources. This study introduces the perspective of psychological ownership and proposes a dual-pathway model of idol–fan psychological ownership and fans’ collective psychological ownership, aiming to uncover the deep psychological foundations that shape the formation of fan enthusiasm.

The results show that perceived control significantly enhances idol–fan psychological ownership. This finding indicates that fans’ sense of control is symbolic, individualized, and emotionally driven. Through visible behaviors such as voting, comment management, and reposting, fans experience a subjective sense of agency that contributes to their idol’s advancement. This aligns with previous research suggesting that social media provides opportunities for individuals to exert some degree of control over their environment (Karahanna et al., 2015; Kwon, 2020).

However, perceived control did not have a significant effect on fans’ collective psychological ownership, which contrasts with prior research on community-based psychological ownership (Kumar, 2019; Lee and Suh, 2015). This discrepancy reveals the fragile and individualized nature of control in fan contexts. Control is fundamentally rooted in platform rules, entertainment agency strategies, and internal group organizational orders, echoing discussions by Zhang et al. (2023), Xiao et al. (2025) regarding algorithmic collective action in fandoms.

Specifically, decisions about collective actions related to idols are often made by core fan groups such as “data teams,” while ordinary fans typically follow instructions and complete assigned tasks to affirm their position within the group. This dynamic makes it difficult for them to develop a shared sense of control over collective norms and goals. Such “structured dependence,” shaped by information asymmetry and organizational hierarchy, reinforces group-based responsibility but limits the transformation of individual perceived control into collective psychological ownership.

As expected, self-investment and intimate interactions positively influenced both idol–fan psychological ownership and fans’ collective psychological ownership. Self-investment is no longer limited to the traditional notion of contributing personal resources in psychological ownership research. Platform mechanisms amplify the visibility of individual actions, and elements such as support posts, trending topics, and fan activities are treated as digital territories that must be protected. Each act of content creation, voting, or fan support becomes a substantive contribution to shaping the idol’s public image (Zhang et al., 2023). Through collective collaboration, individual intention becomes embedded in the idol’s production process, making ownership not merely a one-directional psychological perception but a form of group emotion, an action logic, and an identity practice (Verkuyten, 2025).

At the same time, platform features such as live-streaming and real-time comment overlays create a sustained sense of emotional presence. High-intensity intimate interactions strengthen fans’ individual feelings of belonging. These findings are consistent with discussions of efficacy and belonging in the psychological ownership formation process (Pierce et al., 2001; Peck and Luangrath, 2023).

Cultural symbol value had significant effects on both forms of psychological ownership, indicating that idols in digital fan culture are not merely information carriers but highly symbolic “cultural products.” Fans use idols as mediums for expressing aesthetics, identity, and personal values, projecting their ideal selves onto them to reduce self-discrepancy (Lee and Suh, 2015). In fragmented information environments, stable and repeatedly mobilizable symbols—such as support colors or fan names—serve as anchors that sustain emotional engagement and group identity.

This finding also aligns with the halo effect discussed in psychological ownership research, in which ownership feelings are strengthened when associated with an idealized other, a celebrity, or a highly valued object (Peck and Luangrath, 2023). The result further complements traditional psychological ownership research by addressing the previously overlooked role of target-object attributes (Krauss and Wienrich, 2025).

From the perspective of Goal-Framing Theory (Lindenberg, 2001; Lindenberg and Steg, 2007), the finding that fan emotionality predicts individual psychological ownership but not collective psychological ownership can be further explained. According to this theory, individual behavior is guided by three core goal frames: the hedonic frame (immediate emotions and pleasure), the gain frame (personal benefits), and the normative frame (group responsibility and moral obligations).

Fan emotionality primarily activates the hedonic frame, which centers on emotional arousal and instant gratification, as well as the gain frame, which emphasizes emotional satisfaction, identity confirmation, and the sense of happiness derived from following idols. Under these conditions, the formation of idol–fan psychological ownership at the individual level is a natural outcome. In contrast, the development of collective psychological ownership depends on the normative frame, which is grounded in group responsibility, norms, and rule compliance. Digital fan communities, however, are characterized by fluidity and the transience of emotions (Arvidsson and Caliandro, 2016). Emotional expression is often fragmented and performative, and fan emotional investment tends to manifest as momentary emotional release and short-lived fervor. Such patterns make it difficult to accumulate stable collective identity resources and to activate the normative frame required for collective psychological ownership.

This study confirms that psychological ownership significantly fuels fan enthusiasm and further uncovers new manifestations of fan enthusiasm within digital media environments. Fan enthusiasm is no longer merely the product of emotional attachment; rather, it has evolved into a form of sustained participation characterized by both emotional intensity and behavioral engagement, driven by psychological ownership. Digital media—through mechanisms such as instant feedback, organized collaborative structures, and platform algorithmic logics—substantially amplifies the motivational and reinforcing effects of psychological ownership on fan enthusiasm. As a result, fans in the digital era display forms of enthusiasm that are more tightly connected, proactive, and even marked by elements of pressure and responsibility, extending recent research on fan engagement, algorithmic labor, and collective action (Xiao et al., 2025; Zhang et al., 2023).

Notably, psychological ownership within digital fan culture may also exhibit darker facets. These include self-denigration, group-based aggression or boycotting, and feelings of shame or exclusion—dynamics that may culminate in extreme forms of enthusiasm (Cocieru et al., 2019; Baradar et al., 2023). These phenomena highlight promising directions for future research.

Finally, the fsQCA results reveal the configurational complexity of the antecedents, thereby supporting Propositions P1, P2, and P3. Configuration S1, which features the shared core conditions of self-investment and intimate interaction along with the shared peripheral condition of perceived control, aligns with the classic formation paths of psychological ownership (Pierce et al., 2003). Even when fan emotionality (S1a) and cultural symbol value (S1b) are low, fan enthusiasm can still emerge as long as these core paths of interaction, investment, and control are activated. Configuration S2 demonstrates that, even if certain variables are nonsignificant in linear models, they may still influence outcomes through synergistic effects when combined with other factors. Although perceived control does not significantly predict collective psychological ownership in SEM, it appears as a peripheral condition in the high-enthusiasm configurations in fsQCA, illustrating the “causal asymmetry” characteristic of configurational analysis (Ragin, 2008). Configuration S3 underscores the importance of fan emotionality, echoing prior research on emotional mobilization and collective affective resonance within fan communities (Zhang et al., 2023). Both S2 and S3 include high idol–fan psychological ownership and high collective psychological ownership, thereby corroborating the mediating effects detected in the SEM model and further confirming Proposition P3.

Overall, these findings highlight the complexity and diversity of fan behaviors and showcase the complementarity between SEM and fsQCA. Together, they provide a more comprehensive explanation of the mechanisms underlying the formation of fan enthusiasm.

### 5.2 Theoretical implications

First, this study introduces psychological ownership as a core explanatory framework for understanding high-intensity enthusiasm, emotional labor, and collective action in digital fan culture. This advances the theoretical boundaries of existing fan studies and contributes to the literature on fan engagement and fan loyalty. Prior research has largely relied on parasocial relationships, emotional attachment, and identity-based explanations to account for fans’ affection, belonging, and participation. However, these frameworks are insufficient for explaining the increasingly prominent, near-“sovereign” behaviors observed in contemporary fan culture—such as large-scale data labor, routinized comment control, monetized voting, and the exclusionary protection of digital territories (e.g., occupying public platforms or resisting “boundary-crossing”).

By incorporating the perspective of psychological ownership, this study reveals why fans do not merely “like” idols but come to perceive them as “ours,” which in turn generates responsibility, protection motives, and sustained investment. This extends recent research on algorithmic labor, fan algorithmic activism, and platform-mediated fan practices (Xiao et al., 2025; Zhang et al., 2023), and provides a more powerful explanatory lens for understanding the emotional intensity, behavioral density, and sense of empowerment that characterize fan participation in contemporary digital environments.

Second, this study extends psychological ownership (PO) theory by empirically distinguishing the formation pathways of individual fan psychological ownership and collective fan psychological ownership. Whereas most prior research has focused on psychological ownership as an individual-level construct, this study demonstrates that the two dimensions follow distinct generative mechanisms. Building on the classic formation routes proposed by Pierce et al. (2001), Lee and Suh (2015), Kwon (2020)—namely investing the self into the target of ownership, having control over the target, and intimately knowing the target—this study incorporates target-object attributes and fan-specific traits. In doing so, it responds to recent calls for greater attention to target attributes as well as individual and group structural factors in PO research (Krauss and Wienrich, 2025; Peck and Luangrath, 2023).

In addition, the finding that perceived control and fan emotionality do not significantly shape collective psychological ownership reveals that, within highly organized and knowledge-asymmetric fan communities, the emergence of PO is shaped by group hierarchy, normative structures, and heterogeneity in emotional responses. This aligns with social identity theory’s emphasis on the group self as a core mechanism in the formation of collective ownership, and further advances research on group dynamics and collective psychological processes (Verkuyten, 2025).

Finally, this study advances the application of multi-method analytical approaches in fan research and offers a new methodological framework for explaining the multiple and complex mechanisms underlying fan behavior. Traditional fan studies often rely on single statistical models, which struggle to capture the nonlinear relationships among psychological, emotional, individual, and group-level factors (Pappas and Woodside, 2021). By combining PLS-SEM and fsQCA, this study integrates a variable-centered perspective with a configuration-centered perspective, revealing both the linear influence pathways of psychological ownership and the ways in which different combinations of conditions jointly drive fan enthusiasm. The complementarity of the two approaches allows for a more comprehensive understanding of heterogeneity and complexity in fan behavior.

In the context of digital fan culture, where individual emotion, algorithmic labor, and organized collective practices intersect, this analytical framework not only strengthens the robustness of empirical findings but also provides an extendable methodological paradigm for future research. It encourages the field to move beyond single-path explanations toward a recognition of the multiple pathways and coexisting mechanisms that shape fan behavior.

### 5.3 Practical implications

The findings of this study offer meaningful practical implications for talent agencies in the idol industry, brand managers, and platform governance actors.

First, operational priorities should shift from generating short-term traffic to cultivating psychological ownership. Talent agencies and brands should move beyond a narrow focus on transient visibility and instead foster a sustained sense of ownership among fans. Rather than relying solely on formal ownership mechanisms such as membership tiers, organizations can design experiences that activate psychological ownership. Examples include providing customized content, opening channels for co-creation such as lyric writing or merchandise design, and enhancing feelings of exclusivity through curated interactions. These strategies can help transform fans from passive spectators into active co-creators.

Second, based on the fsQCA configurational results, operators should adopt differentiated strategies tailored to distinct fan types.

For interaction-oriented fans (Configuration S1), platforms should provide efficient community tools, task-distribution systems, and group-based honor incentives that allow these fans to gain fulfillment through participation and social interaction.

For control-oriented fans (Configuration S2), whose core need is influence, agencies should establish dedicated communication channels for this group (often including “career-focused fans”) and grant them a degree of genuine control within appropriate boundaries, such as non-core decisions, while strengthening their voice and status within the fan community.

For emotion-driven fans (Configuration S3), platforms and agencies should offer highly affective, in-depth content such as vlogs and behind-the-scenes narratives to reinforce their emotional connection with both the idol and the fan community.

Third, while cultivating fan identification and a sense of psychological ownership, operators and platforms must also remain alert to the psychological and social risks that may arise, and strengthen cultural guidance and mental well-being functions. Platforms and agencies should balance fans’ desire for control and their need for belonging by implementing transparent rules, tiered feedback mechanisms, and rational discussion spaces, thereby reducing irrational behaviors such as comment manipulation or fan-initiated trolling.

Fan communities should also be recognized as important sites for youth socialization and emotional learning. Through co-created public welfare initiatives, rational support practices, and issue-based advocacy, psychological ownership can be transformed into social responsibility and collective positive energy. This approach not only supports fans’ emotional regulation but also improves the public image and social value of idol culture.

### 5.4 Limitations and future research suggestions

This study, being exploratory in nature, has certain limitations. First, it is a cross-sectional study conducted at a specific point in time, whereas the relationship between fans and idols is dynamic. Previous research has highlighted the continuity of fan identity across different stages of the life course. Future studies could adopt a longitudinal approach to track fan behavior over time, elucidating the psychological mechanisms underlying sustained identity and the lifecycle of psychological ownership within a temporal dimension.

Second, it is important to examine the effects of additional moderating factors in the model, such as perceived heterogeneity and group support dynamics. Furthermore, while this study primarily focuses on the positive effects of psychological ownership, it acknowledges the existence of negative impacts. Future research could explore the negative influences of psychological ownership in contexts such as idol moral failings and “exclusive fandom.”

## Data Availability

The datasets presented in this study can be found in online repositories. The names of the repository/repositories and accession number(s) can be found in the article/supplementary material.

## Footnotes

1
https://www.wjx.cn/
